# Bone marrow-derived myeloid cells transiently colonize the brain during postnatal development and interact with glutamatergic synapses

**DOI:** 10.1016/j.isci.2024.110037

**Published:** 2024-05-21

**Authors:** Micaël Carrier, Marie-Ève Robert, Marie-Kim St-Pierre, Fernando González Ibáñez, Elisa Gonçalves de Andrade, Audrée Laroche, Katherine Picard, Haley A. Vecchiarelli, Julie C. Savage, Éric Boilard, Michèle Desjardins, Marie-Ève Tremblay

**Affiliations:** 1 Axe neurosciences, Centre de recherche du CHU de Québec-Université Laval, Québec, QC G1V 4G2, Canada; 2 Département de psychiatrie et de neurosciences, Faculté de médecine, Université Laval, Québec, QC G1V 0A6, Canada; 3 Division of Medical Sciences, University of Victoria, Victoria, BC V8P 3E6, Canada; 4 Département de médecine moléculaire, Faculté de médecine, Université Laval, Québec, QC G1V 0A6, Canada; 5 Département de microbiologie et immunologie, Faculté de médecine, Université Laval, Québec, QC G1V 0A6, Canada; 6 Department of Physics, Physical Engineering and Optics, Université Laval, Québec City, QC G1V 0A6, Canada; 7 Oncology Division, Centre de recherche du CHU de Québec, Université Laval, Québec City, QC G1V 4G2, Canada; 8 Department of Biochemistry and Molecular Biology, The University of British Columbia, Vancouver, BC V6T 1Z4 Canada; 9 Department of Neurology and Neurosurgery, McGill University, Montréal, QC H3A 0G4 Canada; 10 Centre for Advanced Materials and Related Technology (CAMTEC), Institute on Aging and Lifelong Health (IALH), University of Victoria, Victoria, BC V8W 2Y2, Canada

**Keywords:** Natural sciences, Biological sciences, Neuroscience, Developmental neuroscience, Cellular neuroscience

## Abstract

Although the roles of embryonic yolk sac-derived, resident microglia in neurodevelopment were extensively studied, the possible involvement of bone marrow-derived cells remains elusive. In this work, we used a fate-mapping strategy to selectively label bone marrow-derived cells and their progeny in the brain (FLT3^+^IBA1^+^). FLT3^+^IBA1^+^ cells were confirmed to be transiently present in the healthy brain during early postnatal development. FLT3^+^IBA1^+^ cells have a distinct morphology index at postnatal day(P)0, P7, and P14 compared with neighboring microglia. FLT3^+^IBA1^+^ cells also express the microglial markers P2RY12 and TMEM119 and interact with VGLUT1 synapses at P14. Scanning electron microscopy indeed showed that FLT3^+^ cells contact and engulf pre-synaptic elements. Our findings suggest FLT3^+^IBA1^+^ cells might assist microglia in their physiological functions in the developing brain including synaptic pruning which is performed using their purinergic sensors. Our findings stimulate further investigation on the involvement of peripheral macrophages during homeostatic and pathological development.

## Highlights

• FLT3^+^IBA1^+^ cells infiltrate the brain in the first 2 weeks of life peaking at day 14
• FLT3^+^IBA1^+^ cells are morphologically distinct from microglia, evolving with time
• FLT3^+^IBA1^+^ cells express TMEM119 and P2RY12 and engulf glutamatergic synapses
• Cells show similar side and forward scatter than marginal microglia

## Graphical abstract

**Figure:**
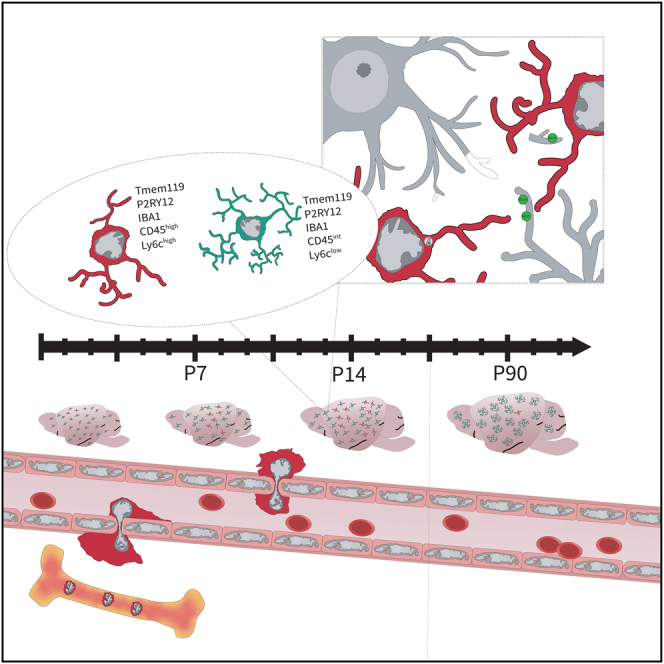

## Introduction

Microglia come from yolk sac’s progenitors that migrate into the brain to fulfill their role as resident immune cells.^1^ In the periphery, macrophage populations have different origins depending on the developmental stage during which they arise (e.g., aorta-gonad-mesonephros, fetal liver, and bone marrow).^2^ Bone marrow-derived cells (BMDCs) are the final source of peripheral immune cells, continuously generating immune cells (e.g., neutrophils and monocytes) from their common progenitors.^3^ BMDCs differentiate into diverse populations based on the microenvironment of the tissue or organ they infiltrate.^4^ Macrophages from the circulation have the ability to infiltrate the brain during environmental challenges like psychological stress^5^ and pathological states including epilepsy.^6^ Even though microglia and BMDCs have different origins, they share multiple characteristics such as homeostatic patrolling and cellular debris clearance.^7^ Other than their role during brain pathology exerted in concert with microglia,^8^ BMDCs have a myriad of functions during homeostasis in the periphery as they provide immune surveillance, secretion of inflammatory factors, adaptive immunity, and inhibition of the immune response to promote tissue repair.^9^^,^^10^

Multiple approaches to discern microglia from peripheral cells have been designed.^11^^,^^12^ However, most of them do not allow to study the brain development, in a homeostatic manner, by examining BMDCs across ages starting at birth. While microglia have been highlighted as a key player in pre- and postnatal neural development,^13^^,^^14^^,^^15^^,^^16^^,^^17^^,^^18^^,^^19^ the possible contribution of BMDCs has remained elusive. Microglia were shown to participate in the maturation of the brain circuitry via the secretion of trophic factors that promote synapse formation.^20^ Microglia also remodel synapse physically via phagocytosis^19^^,^^21^^,^^22^^,^^23^ and trogocytosis,^24^ as well as synaptic stripping (physical separation of pre- and post-synaptic elements).^25^^,^^26^^,^^27^ The identification of these microglial roles has been essential to the current understanding of how brain development takes place.

To study the roles of BMDCs during neurodevelopment, we set out to track the BMDCs in the developing mouse using a Cre driver under the expressions of the BMDC transcription factor fms related receptor tyrosine kinase 3 (FLT3) also known as cluster of differentiation 135 (CD135).^12^ The Flt3-Cre; RFP-flox reporter utilizes the transcription factor Flt3 expressed by short-term hematopoietic stem cells including lymphoid and myeloid progenitors. This resulting construct is able to track neutrophils, monocytes, lymphocytes, and NK cells. This was paired with an immunohistochemistry for ionized calcium-binding adapter molecule 1 (IBA1) when possible to investigate specifically monocytes.^28^ Our initial imaging revealed the presence of BMDC (FLT3^+^IBA1^+^ cells) in the brain during the first and second weeks of life. This finding complements and extends on a previous report which found BMDCs during early development in the postnatal day (P)1–2^29^ brain. We thus set out to (1) investigate among the brain parenchyma FLT3^+^IBA1^+^ cells density and morphology compared to FLT3^-^IBA1^+^ microglia using complementary fluorescent microscopy approaches (e.g., slide scanning and confocal), (2) investigate their ultrastructural features and interactions with neighboring elements including the vasculature and synapses using scanning electron microscopy, and (3) determine their physiological profile using flow cytometry in order to provide novel insights into their roles exerted in concert with microglia within the developing brain.

## Results

### FLT3^+^IBA1^+^ cells infiltrate the brain in the first two weeks of life, peaking at postnatal day 14 where they present a distinct morphological profile compared to FLT3^-^IBA1^+^ microglia

**Figure 1:**
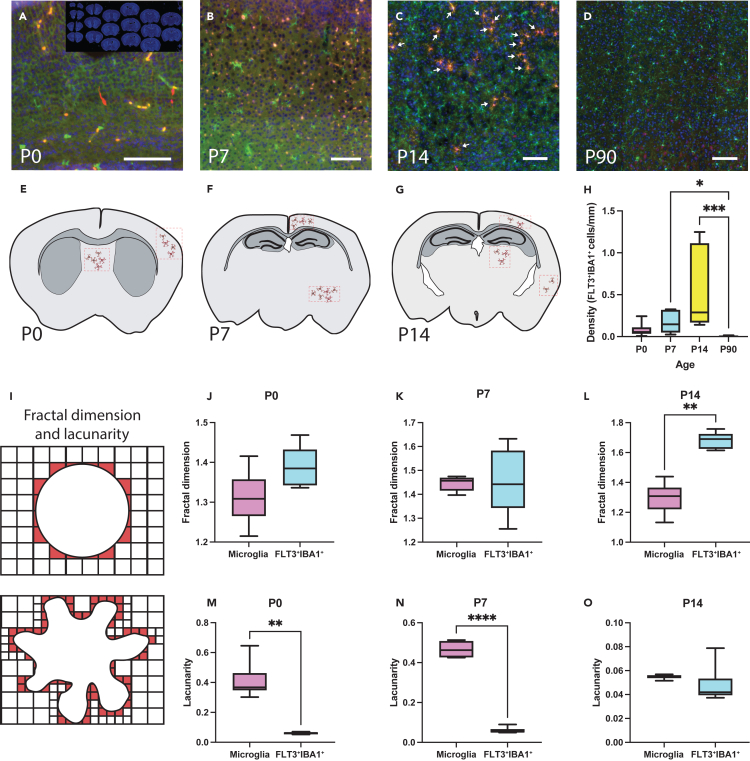
Infiltrating BMDC density in the developing brain and their fractal-based morphology analysis (A–D) Representative images of infiltrating BMDC at P0, P7, P14, and P90 with representative image (inset) of the serial sections disposition used in the experiment, FLT3^+^IBA1^+^ cells are highlighted with white arrows. (E–G) Representative illustrations of the localization of some of the clusters of FLT3^+^IBA1^+^ cells found in the brain in P0, P7, and P14 animals. P90 animals did not present any FLT3^+^IBA1^+^ cell clusters. (H) Non-parametric Kruskal-Wallis statistical analysis of the density of BMDC in the brain across multiple ages (n = 6–8 animals). (I) Illustrations explaining the fractal dimension and lacunarity analysis. (J–L) Paired Student’s t test statistical analysis of the fractal dimension morphology analysis on BMDCs in the brain across ages compared to microglia (*n* = 12–15 cells per age, *N* = 7 animals). (M–O) Paired Student’s t test statistical analysis of the fractal lacunarity morphology analysis on FLT3^+^IBA1^+^ cells in the brain across ages compared to FLT3^-^IBA1^+^ microglia (*n* = 12–15 cells per age, *N* = 7 animals). *p* < 0.05 was considered as statistically significant (∗ = *p* < 0.05, ∗∗ = *p* < 0.01, ∗∗∗ = *p* < 0.001, ∗∗∗∗ = *p* < 0.0001). Scale bar, 20 μm.

Peripheral macrophages were previously found to invade the mouse brain mainly during environmental challenges and pathology^6^ with one investigation finding them during development.^29^ To study FLT3^+^IBA1^+^ cells in the postnatal brain, we used the constitutive FLT3-Cre/RFP-flox mouse model in which FLT3^+^IBA1^+^ cells, but not microglia, are positive for FLT3 and thus easily tracked by their red fluorescence throughout life. We sought to map these FLT3^+^IBA1^+^ cells during normal brain development across different stages (Figures 1A–1D inset) (P0, 7, 14, 90). By imaging serial sections representing the whole brain using a slide scanner, we found that FLT3^+^IBA1^+^ cells are observed at P0 (Figure 1A) but also at P7 (Figure 1B) and P14 (Figure 1C), but not at adulthood (P90, Figure 1D), indicating that FLT3^+^IBA1^+^ cells reside in the rodent brain during the first weeks of life. FLT3^+^IBA1^+^ cells in the brain tended to form clusters that we identified in a few regions at Bregma level 4.76 mm (e.g., lateral septal nucleus and somatosensory cortex) at P0 (Figure 1E), Bregma level 2.12 mm (e.g., motor cortex and lateral hypothalamic area) at P7 (Figure 1F) and the visual cortex (Bregma level 1.64 mm) at P14 (Figure 1G). Specific regions were illustrated for reference (Figures 1E–1G). The density of FLT3^+^IBA1^+^ cells was determined for the entirety of the serial section samples revealing that these cells become gradually more abundant with age through P0-P14 (Figure 1H). Notably, there was a significant peak of FLT3^+^IBA1^+^ cells density observed at P14 (Figure 1H) which coincides with an important window of brain maturation in mice where synaptic pruning takes place.^17^^,^^22^^,^^30^ Moreover, FLT3^+^IBA1^+^ cells were not detected in the P90 brain, contributing to the body of evidence on the lack of peripheral cells in the healthy young adult mouse brain.^9^^,^^31^ The visual cortex is a region of high plasticity in the postnatal brain,^22^^,^^23^^,^^32^ which could explain why we found abundant clusters of FLT3^+^IBA1^+^ cells. Therefore, we decided to use this region for our morphological characterization.

FLT3^+^IBA1^+^ from the slide scanning data were assessed for morphology using the fractal dimension (Dmoy) and lacunarity (Figure 1I) workflow.^33^^,^^34^ The analysis of fractal dimension revealed a tendency for more elevated values in FLT3^+^IBA1^+^ cells versus microglia at P0 (Figure 1J) and P7 (Figure 1K), which reached significance at P14 (Figure 1L), indicating that FLT3^+^IBA1^+^ cells have a more complex geometrical morphology compared to microglia in the brain parenchyma^35^ (i.e., higher fractals dimension indicate a more complex geometrical morphology which could be interpreted as needing more and smaller pixels to trace the perimeter of the cell). FLT3^+^IBA1^+^ cells also exhibited lower lacunarity (i.e., less homogenous spacing between processes) compared to FLT3^-^IBA1^+^ microglia (Figures 1M–1O). These findings indicate that FLT3^+^IBA1^+^ cells and FLT3^-^IBA1^+^ microglia display distinct morphologies across the first postnatal weeks.

### FLT3^+^IBA1^+^ cells evolve morphologically across early postnatal development, express the microglial markers P2RY12 and TMEM119, and interact with VGLUT1 synapses at P14

**Figure 2:**
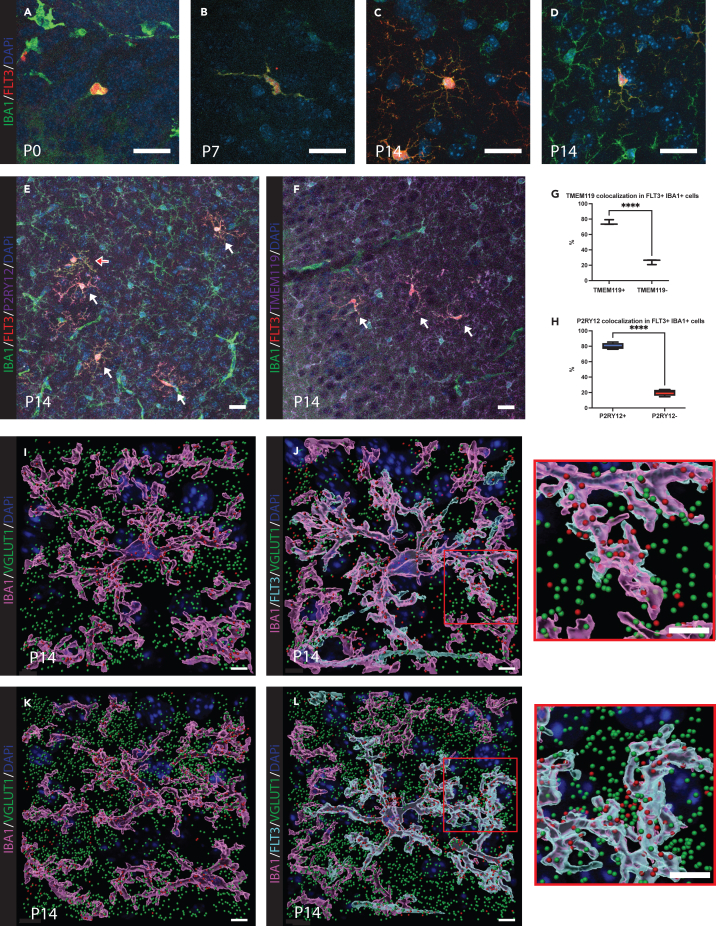
BMDCs are morphologically distinct from microglia, yet they express the microglial markers P2RY12 and TMEM119 and prune VGLUT1 synapses (A–D) Representative images using confocal microscopy of the FLT3^+^IBA1^+^ cells across ages. (E) Representative image of the P2RY12 staining in confocal microscopy at P14, FLT3^+^IBA1^+^ cells are highlighted with white arrows. (F) Representative image of the TMEM119 staining in confocal microscopy at P14, FLT3^+^IBA1^+^ are highlighted with white arrows. (G) Non-parametric Student’s t test with Welch’s correction statistical analysis of the cells with TMEM119 colocalization over the cells non-colocalized with TMEM119 in the brain at P14 (*n* = 12–15 cells, *N* = 3 animals). (H) Non-parametric Student’s t test with Welch’s correction statistical analysis of the cells with P2RY12 colocalization over the cells non-colocalized with P2RY12 in the brain at P14 (*n* = 12–15 cells, *N* = 4 animals). *p* < 0.05 was considered as statistically significant (∗∗∗∗ = *p* < 0.0001). Scale bar, 20 μm. (I and K) Representative image of the VGLUT1 staining on FLT3^-^IBA1^+^ microglia in confocal microscopy at P14 (*n* = 1–3 cells, *N* = 5 animals) Scale bar, 5 μm. (J and L) Representative image of the VGLUT1 staining on FLT3^+^IBA1^+^ cells in confocal microscopy at P14 (*n* = 1–3 cells, *N* = 5 animals). Scale bar, 5 μm. Zoom-in was made to highlight the pruning event.

Microglial morphology is highly plastic, constantly changing during brain development and throughout life.^14^^,^^36^^,^^37^ Fractal analysis highlighted the differences in morphological features between FLT3^+^IBA1^+^ cells and FLT3^-^IBA1^+^ microglia during early postnatal development. To examine at higher resolution the morphological features of FLT3^+^IBA1^+^ cells, we imaged these cells across multiple time points using confocal microscopy. Confocal imaging of FLT3^+^IBA1^+^ cells at P0 (Figure 2A), P7 (Figure 2B) and P14 (Figure 2C). and microglia at P14 (Figure 2D) was performed in the visual cortex (Figures S1A–S1P). At P0, both FLT3^+^IBA1^+^ cells and FLT3^-^IBA1^+^ microglia displayed an ameboid morphology, characterized by few ramifications (Figure 2A). In FLT3^-^IBA1^+^ microglia, this morphological state was previously associated with a high migratory activity in the mouse and zebrafish brain.^38^^,^^39^ At P7, FLT3^+^IBA1^+^ cells did not bear many secondary processes branches (Figure 2B), suggesting a morphological transition between the FLT3^+^IBA1^+^ cells seen at P0 and those at P14 or a ramified morphology adopted by recently infiltrating FLT3^+^IBA1^+^ cells at P14.^6^^,^^38^ By P14, FLT3^+^IBA1^+^ cells were highly ramified, and their process morphology could be classified as highly irregular (i.e., processes having sharp curves) (Figure 2C). We also observed in these FLT3^+^IBA1^+^ cells many secondary angular processes, contrary to FLT3^-^IBA1^+^ microglia at the same developmental stage (Figures 2C and 2D).

Previous reports revealed an age-dependent expression of TMEM119 and P2RY12 in mouse microglia at P14 and in mouse liver macrophages^40^ and the brain at P24.^29^ We thus examined whether the FLT3^+^IBA1^+^ cells detected at P14 expressed P2RY12, which plays a key role in synaptic plasticity within the visual system^32^ (Figures 2F and S1V–S1Z) and TMEM119 (Figures 2E and S1Q–S1U). Immunofluorescence staining for these markers revealed that FLT3^+^IBA1^+^ cells express TMEM119 in 76% (mean ± SEM = 59.77 ± 2.78%) (Figure 2G) of the cells and P2RY12 in 80% (mean ± SEM = 50.57 ± 2.74%) of the cells (Figure 2H) in the visual cortex at P14. The expression of microglial markers suggests their potential to aid microglia with their homeostatic function in the developing brain.

Microglia are known to prune synapses during early postnatal development. We tested whether this ability was shared by FLT3^+^IBA1^+^ cells. We conducted staining for VGLUT1 synapses and examined the localization of the labeled puncta. FLT3^+^IBA1^+^ cells presented numerous VGLUT1^+^ puncta inside or in close interaction with them in the P14 mouse motor cortex (Figures 2I and 2K), as for FLT3^-^IBA1^+^ microglia (Figures 2J, 2L, and S2A–S2D).

### FLT3^+^ macrophages and FLT3^-^ microglia show distinct ultrastructural features and interactions within the visual cortex neuropil at P14

**Figure 3:**
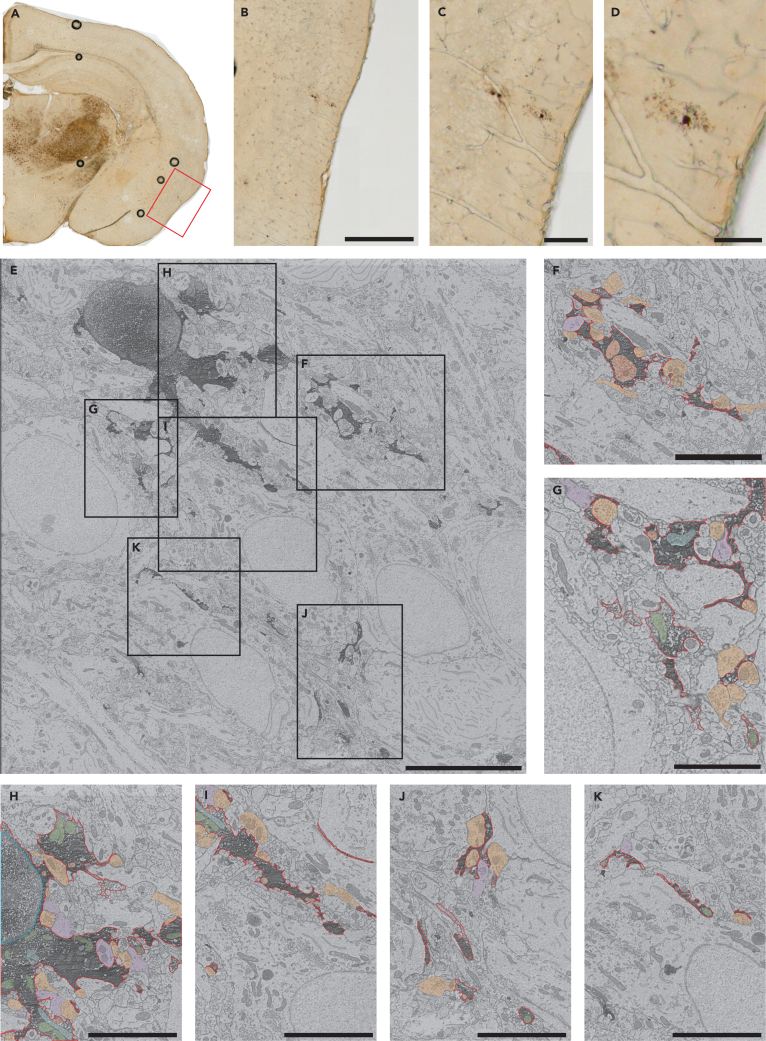
Light and scanning electron microscopy imaging of the FLT3^+^ cells in the developing brain parenchyma presenting thin processes contacting and engulfing synaptic elements (A) Brightfield images of FLT3^+^ cells in the brain parenchyma at P14 with zoomed in images of a ramified FLT3^+^ cell with macrophage features in (B–D). (E) Representative image of a FLT3^+^ cell in the piriform cortex of a P14 male mice with zoomed in images in (F–K). (F–I) Engulfed pre-synaptic axon terminals within FLT3^+^ cell body and processes. Altered mitochondria are also observed within the cytoplasm of the FLT3^+^ cell. (J and K) FLT3^+^ processes are shown directly juxtaposing a neuronal cell body. Red outline = cytoplasmic membrane, blue outline = nuclear membrane, orange pseudo-coloring = pre-synaptic axon terminal, purple pseudo-coloring = post-synaptic dendritic spine, red pseudo-coloring = phagosome, green pseudo-coloring = non-altered mitochondria, blue pseudo-coloring = altered mitochondria. (B) Scale bar, 200 μm; (C) Scale bar, 50 μm; (D) Scale bar, 25 μm; (E) Scale bar, 10 μm; (F-K) Scale bar, 5 μm.

To gain insights into their possible roles, we next investigated the ultrastructure of FLT3^+^ macrophages, examining FLT3^+^ cell bodies and processes presenting features of macrophages within the visual cortex of P14 mice at Bregma level 1.64 mm. Stained brain sections were imaged using brightfield microscopy before their processing for correlative scanning electron microscopy to identify cells of interest (Figures 3A–3D). FLT3^+^ were observed in the brain parenchyma both far (more than 10 μm) from the first capillary vessel (Figure 3E) but also nearby (less than 10 μm) from the closest capillary vessels (Figure S3D). The FLT3^+^ overall morphology (e.g., acute cytoplasmic angles, angular hyper-ramified processes, Figure S1A) was distinct from FLT3^-^ microglia examined in the same samples (Figure S1A) and from microglia typically seen in the developing brain^22^^,^^41^^,^^42^ and monocytes in the circulation (Figures S1B and S1C).^43^ Intracellular contents and parenchymal interactions of FLT3^+^ cells were examined to provide insights into the possible function of these cells during normal postnatal development. At P14, FLT3^+^ cells possessed several mitochondria (Figures 3E–3K), some of which were altered in their cristae and outer membrane structure (Figures 3G–3I), an ultrastructural feature that suggests a possible up-regulation in the bioenergetic production of these cells.^44^

In addition, we observed several phagosomes internalized within the cell body (Figure 3H) and processes (Figures 3F–3J and 3I) of FLT3^+^ cells. These phagosomes contained mostly axon terminals recognized by their synaptic vesicles (pseudocolored in red in Figures 3F–3I). Supporting the extensive interactions between FLT3^+^ cells and synaptic elements, FLT3^+^ cells were often in direct contact with axon terminals and dendritic spines (pseudocolored in orange and purple, respectively, in Figures 3F–3K). Processes of FLT3^+^ cells also directly juxtaposed neuronal cell bodies (Figures 3I–3K), which suggests their involvement in physical separation of pre-synaptic innervations onto neuronal cell bodies via synaptic stripping,^45^^,^^46^^,^^47^ not uncommon for microglia at this age.^48^ FLT3^+^ cell bodies also occupied satellite positions (cell-to-cell contact; Figures 1E and S1D), this highlights a close relationship between neurons and FLT3^+^ cells which might affect neuronal activity as it has been shown for microglia.^49^^,^^50^

### Infiltrated FLT3^+^ cells keep their peripheral molecular signature in the brain

BMDC blood molecular signatures vary depending on the context, as shown by flow cytometry experiments in diseases including amyotrophic lateral sclerosis, and following BMDC engraftment in the mouse brain.^51^^,^^52^^,^^53^^,^^54^ Nevertheless, BMDCs were reported to maintain their identity once they enter the mouse brain.^52^^,^^55^ FLT3^+^ cells in the blood of wild-type C57BL/6J mice were first assessed for their expression of the leukocyte markers CD45, CD115, and GR1. As a control, we also examined the expression of the red fluorescent protein (RFP) which is continually produced in the progeny following the expression of FLT3 in the progenitor cells of our fate mapping system. CD45^high^CD115^high^GR1^high^ and GR1^low^ FLT3^+^ cells from the C57BL/6J mice at P14 and P180 did not present any RFP^+^ cells (Figure S4A). FLT3; cre CD45^high^ FLT3^+^ cells were also successfully separated into GR1^high^ and GR1^low^ populations, which were both FLT3^+^(RFP) at P14 and P90 (Figure S4B), thus validating the effectiveness of the construct. FLT3^+^ cells (RFP) were also observed using epifluorescence in various other organs notably the spleen, liver, and kidney (Figure S4C).

**Figure 4:**
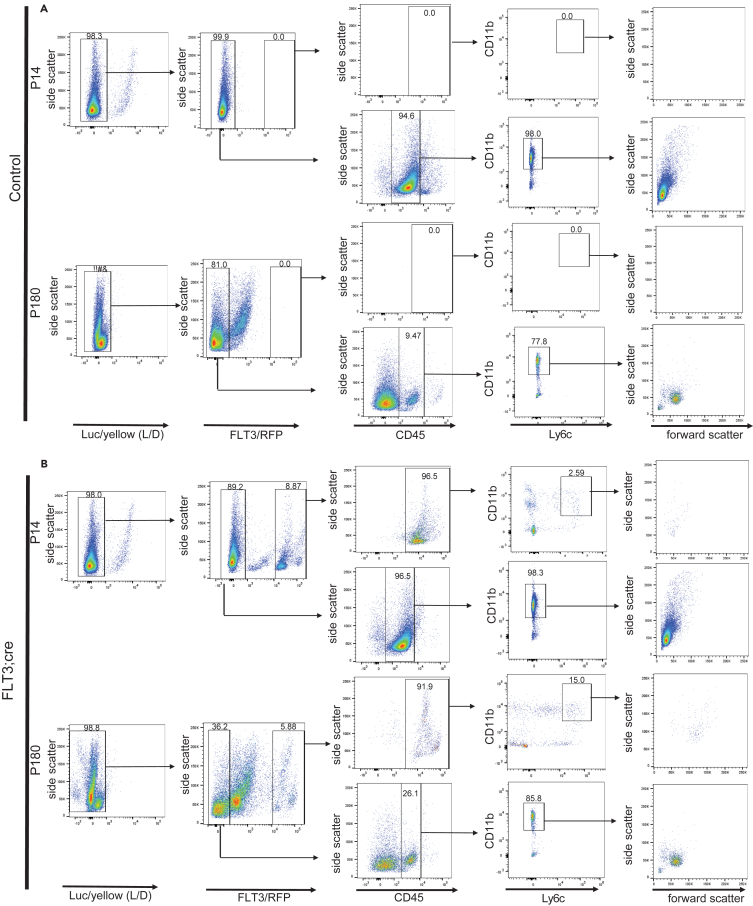
Infiltrating FLT3^+^ cells exhibit a distinct molecular signature through the expression of peripheral proteins including CD45, Ly6c and CD11b (A) Representative flow cytometry analysis of the infiltrating FLT3^+^ cells in the control P14 and P90 mousebrain (*n* = 6 animals). (B) Representative flow cytometry analysis of the infiltrating FLT3^+^ cells in the FLT3; cre P14, and P90 mouse brain (*n* = 6 animals). Numbers represent percentage from parent gate (arrow).

Next, we sought to provide additional molecular insights into the identity of FLT3^+^ cells once they are in the brain parenchyma. In the adult, whole brain isolates from C57BL/6J mice revealed, as expected, a lack of FLT3^+^ cells (RFP)^56^ (Figure 4A). In the P14 control and the FLT3; cre animal, we can see that FLT3^-^ microglia have a diverse side and forward scatter and a more uniform morphology than at P180 (Figures 4A and 4B). In the FLT3; cre animal, FLT3, CD45^high^, Ly6c^+^ cells showed similar side and forward scatter than the marginal FLT3^-^ microglial population while being different from the core population (Figure 4B). Furthermore, FLT3^+^ cells at P180 showed increased forward scatter, adopting a clear morphological difference to FLT3^-^ microglia (Figure 4B).

## Discussion

Our investigation using multi-modal imaging and cellular/subcellular and intercellular characterization of FLT3^+^IBA1^+^ cells during their transient appearance in the developing brain shows that peripheral cells may participate in brain development. These findings build upon previous reports showing the presence of BMDCs in the developing brain. Using large brain samples paired with slide scanning, our results support the contentious idea of peripheral cell infiltration in the developing brain in the first two postnatal weeks, a period where BMDCs were observed.^29^ Using the constitutive cre; lox system labeling all FLT3^+^ cells and their progeny, we found a significant peak of infiltration at P14 using slide scanning. As suggested by correlative light and electron microscopy, FLT3^+^ cells are able to internalize synapses and display a hyper-ramified morphology at P14.^42^ The majority of FLT3^+^IBA1^+^ cells also express TMEM119 and P2RY12, the latter of which is known to be involved in ATP sensing notably modulating synaptic pruning in microglia.^32^^,^^57^^,^^58^ P2RY12 is also known for stimulating microglial activity.^59^^,^^60^ Molecular investigation using flow cytometry further corroborated the peak of FLT3^+^ cells in the brain at P14. These findings place FLT3^+^IBA1^+^ cells as a potential player to help microglia during development. Using animal models where microglia are ablated, microglia were shown to be extensively involved in synapse remodeling.^19^^,^^57^ The FLT3^+^IBA1^+^ cells increase in density followed a similar growth curve as microglia, which were shown to increase significantly in the first 14 postnatal days in mice,^61^ suggesting that FLT3^+^IBA1^+^ cells are complementing microglial number and function in physiological conditions.

Analysis of microglial morphology provides insights into their roles and states.^26^^,^^37^^,^^42^^,^^62^^,^^63^^,^^64^ Similar to microglia,^65^^,^^66^ we showed that FLT3^+^IBA1^+^ cells adopt a distinct migratory morphology (very few processes) early on and later an actively phagocytic morphology (highly ramified) and ultrastructure. Morphological changes between P0 and P7 suggest that FLT3^+^IBA1^+^ cells start from an ameboid shape to adopt a microglia-like morphology, something typically seen after at least 2 days of infiltration in an animal model of epilepsy to support microglia in their pruning endeavors.^67^ Thus, infiltration must have happened in the few first days of life or right before the animals were born. More specifically, the phagocytic phenotype we observed in FLT3^+^ cells in the visual cortex correlates with the observations of microglia in the mouse visual cortex where they are shown to be highly involved.^22^^,^^32^^,^^68^^,^^69^ This process was revealed to be dependent on the P2RY12 receptors in microglia^32^ also expressed by FLT3^+^IBA1^+^cells, suggesting a similar function for FLT3^+^IBA1^+^ cells relying on their sensing activity.^22^ It was previously shown that the expression of TMEM119 is rather variable before P20 in mouse microglia, with less than 80% of microglia expressing TMEM119 before P20.^70^ P2RY12 expression was also shown to vary with age and sex in mice.^71^ This goes in line with our finding that a proportion of FLT3^+^IBA1^+^ cells do not express these markers at this time point, which suggests a heterogeneous population or a delayed expression (cells which might have infiltrated later). Using electron microscopy, we further showed that FLT3^+^ cells interact with synaptic elements and phagocytosed pre-synaptic elements. These presynaptic elements were potentially VGLUT1 synapses shown in confocal microscopy to be inside or in close interaction with FLT3^+^IBA1^+^ cells. This new role is relevant as alterations in the number of VGLUT1 boutons are seen in patients with schizophrenia^72^ and epilepsy.^73^ FLT3^+^IBA1^+^cells could thus be involved. As the glutamatergic system is a major excitatory pathway in the brain, its proper maturation is crucial.^74^ In parallel, FLT3^+^IBA1^+^ cell mitochondria showed ultrastructural signs of high cellular metabolism (e.g., disrupted cristae and degraded outer membrane). A similar result was observed during microglial phagocytosis, a process partly controlled by their mitochondrial activity in adult mice.^75^ A previous study has suggested that mitochondrial length also influences microglial activity.^76^ Deficits in proper mitochondrial control over the phagocytic process can lead to pathology.^75^

Because of their peripheral origin, BMDCs have shown potential for cellular therapy compared to microglia.^77^ Following intravenous engraftment in mice, BMDCs were shown to acquire some microglial features such as ramification, longevity, and clonal expansion but keep most of the BMDC features.^55^ Chimeric mouse models also revealed brain infiltration of BMDCs following microglial depletion together with distinct BMDC identity compared to microglia.^52^ Our model supported previous studies that found the presence of ramifications on infiltrating FLT3^+^IBA1^+^ cells in the healthy^29^ and the pathological brain.^6^ When we investigate the change using flow cytometry, we saw that FLT3^+^Ly6c^+^ cells at P14 show a morphology similar to some FLT3^-^IBA1^+^ microglia at the same age, suggesting that FLT3^+^IBA1^+^ cells adopt the specific morphology of the microglia they are helping in the brain. Microglial role-oriented morphological diversity has gained research interest recently.^36^ Flow cytometry was not precise enough to show clear morphological difference between FLT3^+^Ly6c^+^ cells and microglia at P14 as we measured in microscopy using the fractal analysis. However, we do see a clear difference at P180. This increased diameter at P180 could come from the fact that these cells are most likely residual circulating and perivascular monocytes that are known to be largely ameboid cells. It is important to note the variability of marker expression found in our flow cytometry analysis, particularly in expression of Ly6c. This variability could come from the developmental dynamism known in neuroimmunology. However, this might be a limitation of this fate-mapping system and should be considered in further use of this mouse model. Newer fate mapping models are now showing effectiveness to track BMDCs in the brain in health and disease.^78^ Subsequent studies using this model or others could explore their role in neurodevelopmental disorders, such as schizophrenia and major depressive disorder.^17^^,^^79^^,^^80^^,^^81^ Their transient presence suggests a potential role in sculpting the developing brain. It is yet to be established if their interaction with microglia in the brain is necessary for proper brain development. As BMDCs play a role in vascular remodeling in injury,^82^ it would be of interest to investigate FLT3^+^IBA1^+^ cells’ ability to influence the developing brain vasculature at P0, P7, and P14.

### Conclusion

We found FLT3^+^IBA1^+^ cells in the brain parenchyma during the two first weeks of the mouse life, able to interact with neurons and perform synaptic pruning notably on VGLUT1 synapses. Our findings offer a novel perspective to study the interaction between the peripheral and central immune systems in the context of disorders such as autism, schizophrenia, and more. Further investigating the role of FLT3^+^IBA1^+^ cells in remodeling of specific synaptic networks and their relationship with neuronal progenitors could be the next step toward using these cells in cellular therapy for neurodevelopmental disorders.

## STAR★Methods

### Key resources table

**Table:** 

REAGENT or RESOURCE	SOURCE	IDENTIFIER
**Antibodies**		

anti-IBA1-635	Wako	Cat#013-26471
Mouse anti-IBA1	Millipore	Cat#MABN92
Rabbit anti-TMEM119	Sigma Prestige	Cat#HPA051870-100UL
Rabbit anti-VGLUT1	Millipore	Cat#AB5905
Alexa Fluor 488 donkey anti-mouse IgG (H+L)	Invitrogen	Cat#A21202
Alexa Fluor 647 goat anti-rabbit IgG (H+L)	Invitrogen	Cat#A21245
Anti-RFP	Jackson ImmunoResearch	Cat#111-066-046

**Biological samples**		

C57BL/6J	Jackson laboratory	Strain #:**000664**

**Chemicals, peptides, and recombinant proteins**		

Fluoromount G	Southern Biotech	Cat#0100-01
VECTASTAIN	Vector Labs	Cat#VECTPK6100
3,3′-Diaminobenzidine tetrahydrochloride	Millipore Sigma	Cat#D5905-210 50TAB
Osmium	EMS	Cat#19190
Potassium ferrocyanide	Sigma-Aldrich	Cat#P9387
Thiocarbohydrazide	Sigma-Aldrich	Cat#223220
Durcupan resin	Sigma Canada	Cat#44610

**Experimental models: Organisms/strains**		

FLT3 Cre;Lox mice	Max Planck Institute	

**Software and algorithms**		

FlowJo	BD	v10.10
Prism software	GraphPad	v9.0.1
Adobe Illustrator	Adobe	2024

### Resource availability

#### Lead contact

Further information and requests for resources and reagents should be directed to and will be fulfilled by the lead contact, Marie-Ève Tremblay (evetremblay@uvic.ca).

#### Materials availability

This study did not generate new unique reagents.

#### Data and code availability

All data can be obtained from the lead contact, provided the request is reasonable.

This study did not generate or produce any original code.

### Experimental model and study participant details

The present study involved the FLT3 Cre;Lox animal model developed by Dr. Thomas Boehm at the Max Planck Institute. They were generated on a C57BL/6J. The transgene only is active in male of this strain, preventing sex or gender study using this model.

### Method details

#### Animals

All experiments were approved and performed under the guidelines of Université Laval’s animal ethics committee and the Canadian Council on Animal Care. The animals were housed at 22-25°C under a 12-h light-dark cycle with free access to food and water. The experiments were conducted on Flt3Cre mice were generated by C. Bleul and provided by T. Boehm and S. E. Jacobsen^12^ on a C57BL/6J background crossed with RFPlox to distinguish microglia (FLT3^-^IBA1^+^) from BMDC (FLT3^+^IBA1^+^) during the developmental period (P0, P7, P14, P90). Experimental animals were generated by the cre;lox system where the FLT3 gene is replaced by a RFP reporter gene.^12^ Only male mice were used due to a lack of transgene’s expression in female animals.^12^

#### Fluorescent immunochemistry (confocal, slide scanner)

All animals were anesthetized with a mixed cocktail of ketamine (80mg/kg) and xylazine (10mg/kg). Whole brains were collected from the animals by performing transcardiac perfusion with ice-cold phosphate buffer saline (PBS) with paraformaldehyde (PFA) (4%) before being post-fixed in PFA (4%) overnight at 4°C (Tremblay et al. 2010). The tissues were then flash frozen with dry ice and conserved at -80°C.

All of the mouse brains were cut into 20 μm-thick coronal sections. P0 and P7 brains were cut with a CM1860 UV cryostat (Leica, Germany) on glass slides at -20°C, whereas P14 and P90 brains were cut with a SM2000R freezing microtome (Leica, Germany) and conserved at -20°C in cryoprotectant solution. Tissues from liver, spleen and kidney were processed in the same way and imaged to ensure that BMDC could be found in these other tissues. The sections of interest were later processed for immunofluorescence staining for (1) IBA1, (2) IBA1 and TMEM119, (3) IBA1 and P2RY12. (4) IBA1 and VGLUT1, P14 and P90 brain sections were rinsed 3 times to remove cryoprotectant and were mounted onto slides in phosphate buffer (PB, 100mM, pH7.4) diluted 1:5 with double-distilled water right before staining.

Because of their delicate nature, staining of the P0 and P7 animals was performed on slides and sections were washed in PBS, quenched in citrate buffer, cooled to room temperature, and treated with NaBH_4_ (0.1%) in PBS 50mM. Slides were then incubated in blocking buffer (Table S1). Primary antibodies specific against IBA1 (EMD Millipore, #MABN92), TMEM119 (Sigma Prestige, #HPA051870-100UL), P2Y12 and VGLUT1 (Millipore, #AB5905) were used next. Sections were subsequently incubated in primary antibodies overnight at 4°C. Sections were next incubated in secondary antibodies (Table S1) for 1.5 hours at 4°C. The secondary antibodies used were Alexa Fluor 488 donkey anti-mouse IgG (H+L) (Invitrogen, #A21202) and Alexa Fluor 647 goat anti-rabbit IgG (H+L) (Invitrogen, #A21245). VGLUT1 staining was done subsequently with an anti-IBA1-635 (Wako, #013-26471) incubation as the antibody is directly linked to the fluorophore (Table S1). All slides were also counter-stained with DAPI, rinsed in PB and mounted with an anti-fading medium (Fluoromount G, Southern Biotech #0100-01) under a glass coverslip.

Whole brain samples (one section every 12 from the end of the olfactory bulb to the start of the cerebellum) from P0, P7, P14 and P90 animals (n= 4-6 animals per group) that were stained for IBA1 were imaged using a V120 (Olympus, Japan) slide scanner to visualize microglia (FLT3^-^IBA1^+^) and peripheral macrophages (FLT3^+^IBA1^+^). Cells were manually counted on the whole sample in a blinded manner and reported on the whole area analyzed ≈800mm^2^.

Confocal imaging was performed in the visual cortex (V1) of P14 mice using sections stained for TMEM119/IBA1 and P2RY12/IBA1 (n=6 animals per condition per staining) using a LSM 800 (Zeiss, Germany). Z-stacks containing 30 images with a step size of 1 μm were acquired at 20X magnification. 15 cells per animal were randomly selected to determine the colocalization percentage between TMEM119 or P2RY12 and FLT3^+^IBA1^+^ cells. A single experimenter blinded to the experimental conditions did the colocalization analysis to prevent possible bias.

For the VGLUT1 investigation, slides were imaged on a Zeiss LSM-880 confocal microscope, (Carl Zeiss AG, Oberkochen, Germany), equipped with an Airyscan 2 detection unit. Z-stacks were acquired with a 63× (aperture 1,4) objective. Z-stacks were made with a step size of 1 μm. 5 cells per animal were randomly selected to determine the colocalization of VGLUT1 in FLT3^+^IBA1^+^ cells and in IBA^+^FLT3^-^. VGLUT1 puncta localization was analyzed using IMARIS (Version 9.5.1, Andor), where VGLUT1 puncta interacting or colocalized with FLT3^+^ or IBA1^+^ signal were highlighted in red.

#### Fractal analysis

Automated morphology analysis was performed on brain BMDC (IBA1^+^/FLT3^+^) and microglia (IBA1^+^/FLT3^-^) from the images acquired by slide scanning fluorescent microscopy. A sample of n=10-15 BMDC cells and n=10-15 microglia from N=4-6 animals per condition were randomly selected in the whole brain of P0, P7 and P14 animals. Whole brains were used to increase the heterogeneity of the sample, thus testing the robustness of the analysis. The area, perimeter, fractal dimension and lacunarity of each cell were measured by fractal analysis with the plugin *FracLac* for ImageJ^33^^,^^34^ (https://imagej.nih.gov/ij/plugins/fraclac/FLHelp/Introduction.htm). In brief, each image contained one cell per field. After drawing a square around the cell, the “Analyze Menu” (https://imagej.nih.gov/ij/docs/menus/analyze.html) was used to measure area and perimeter. Then, the image was converted into a binary to extract the outline. Fractal dimension and lacunarity were extracted based on the cell’s contour, with a summary of the calculations available in the plugin’s reference guide (https://imagej.nih.gov/ij/plugins/fraclac/FLHelp/StartUpScreen.htm). In summary, the fractal dimension is an index for the complexity of the cell’s morphology, increasing in proportion to the repetition of a scale-invariant pattern, thus, the pixel detail.^33^ Similarly, the lacunarity refers to the gaps in the image, the more heterogenous they are, the higher the lacunarity.^33^ Fractal dimension and lacunarity are complementary, the first being particularly sensitive to morphology in whole cells, and the latter features such as soma size and process length.^33^

#### Correlative light and electron microscopy

Mice were anesthetized and transcardially perfused with PBS and acrolein (3.5% in PB 100 mM). The brains were extracted and post-fixed in 4% PFA for 2 hours as previously described.^83^ After being rinsed in ice-cold PBS, 50 μm-thick coronal sections of the brains were cut on the vibratome. The sections were stored at -20°C in cryoprotectant.

Sections from the Bregma level 1.64 mm were selected as it was found to have many BMDC cluster in slide scanner mapping. Sections were rinsed in PBS, quenched with 0.3% H_2_O_2_ and treated with NaBH_4_ (0.1%). Sections were then incubated in blocking buffer and incubated in primary antibody against RFP (1:5000, MBL #PM005, 043). The incubation with the primary antibodies was done overnight at 4°C. Sections were then incubated with the secondary antibody Biotin-SP-conjugated Fragment goat anti-rabbit (1:1000, Jackson ImmunoResearch, #111-066-046) for 1.5 hours at room temperature. The sections were washed in Tris-buffered saline (TBS) 0,05M and incubated in avidin-biotin solution (1/100 in TBS; VECTASTAIN ®, Vector Labs, California, USA, Cat# VECTPK6100) for 60 minutes. They were developed in DAB with 0.015% H_2_O_2_ (DAB, 0.05%, Millipore Sigma, Massachusetts, USA, D5905-210 50TAB) for 3 minutes. To examine the ultrastructure of these cells in light and electron microscopy. stained sections of brain were imaged in brightfield microscopy to identify the DAB-stained RFP+ cells. The location of the cells of interest was noted by imaging the brain section using a brightfield microscope (10x, Zeiss) to guide the electron microscopy investigation (Figures 3A and S1D).

Sections with DAB-stained RFP^+^ cells were post-fixed 1 hour with 2% osmium (EMS, Pensylvannia, USA, Cat#19190) and 1.5% potassium ferrocyanide (Sigma-Aldrich, Ontario, Canada, Cat#P9387). Then, sections were incubated for 20 minutes with 1% thiocarbohydrazide (TCH, diluted in double-distilled water; Sigma-Aldrich, Ontario, Canada, cat# 223220) and a second time for 30 minutes with 2% osmium in double-distilled water. Sections were dehydrated with increasing concentrations of ethanol and washed with propylene oxide. They were left in Durcupan resin (20 g component A, 20 g component B, 0.6 g component C, 0.4 g 227 11 component D; Sigma Canada, Toronto, cat# 44610) for 24h at room temperature before being transferred between two fluoropolymer sheets (ACLAR®, Pennsylvania, USA, Electron Microscopy Sciences, cat# 50425-25). The sheets with the resin-embedded sections were incubated at 55-60°C for 72 hours to let the resin polymerize.

The areas of interest were excised from each fluoropolymer sheet, glued on the top of a resin block and incubated again at 55-60°C overnight. The tissues were then cut in 70 nm-thick ultrathin sections with an ARTOS ultramicrotome (Leica, Germany) and collected onto a silicon wafer. Whole sections were mounted on a stud to be faced and mapped with a scanning electron microscope (Figure S1D). Sections were imaged on a Crossbeam 350 scanning electron microscope (Zeiss, Germany) at resolutions of 25 nm/pixel for the imaging of the whole parenchyma and 5nm/pixel for the intracellular and parenchymal interaction of FLT3^+^ microglial cells. Qualitative analysis and pseudo-coloring were done by an experienced experimenter on Adobe Illustrator (Abode, United States). FLT3^+^ cells were identified by the electron-dense DAB immunoprecipitation. Pre-synaptic elements were positively identified by the presence of vesicles while post-synaptic elements possessed an electron-dense postsynaptic density and were found adjacent to a pre-synaptic element.^83^^,^^84^^,^^85^ Mitochondria were ultrastructurally characterized by their double membrane, electron-dense appearance and the presence of cristae.^83^^,^^86^^,^^87^ Altered mitochondria, which are ultrastructural signs of cellular stress, were positively identified based on the disruption of their cristae (visually observed by an electron-lucent space within the mitochondria) or the degradation of the outer membrane (visually identified by an electron-lucent dilation between the outer and inner membrane).^43^^,^^87^ Phagosomes were identified by their defined outline containing cellular material (e.g., pre-synaptic element).^83^^,^^88^

#### Flow cytometry (FACS)

Mice were transcardially perfused with cold 0.9% saline. Whole brains were collected and broken down in a 1X base buffer solution (BB) containing DNAse 1 (100 μg/mL), MgCl_2_ (1 mM) and Accutase (0.5X). The suspensions were incubated overnight at 4°C. The tissue pieces were homogenized in 1X BB by gentle pipetting, and the tubes were then centrifuged at 300xg for 8 minutes at 4°C. After removing the supernatant, the pellets were resuspended in 37% Percoll solution and spun down at 600xg for 20 minutes at room temperature (accelerated 5 and decelerated 1). The pellets were purified and resuspended in BB to remove myelin and clumps and centrifugated at 400xg for 5 minutes at 4°C. Finally, cells were resuspended in 1X HBSS to remove the proteins present in the buffer and centrifuged again at 400xg for 5 minutes at 4°C. After being resuspended in Yellow Live/Dead staining solution, the cells were washed and incubated for 10 minutes on ice in blocking antibody. Extracellular antibodies(CD11b-BV421 (1:100; BioLegend, 101251, clone M1/70), , LY6C-FITC (1:100; BioLegend, 128005, clone HK1.4), CD45-PerCP.Cy5.5 (1:100; (BioLegend, 103132, clone 30-F11), Ly-6G/Ly-6C (Gr-1) (1:100; (BioLegend, 108405, clone RB6-8C5)), CD115 (CSF-1R) (1:100; (BioLegend, 135505, clone AFS98)) , were added, and the cells were incubated again for 30 minutes on ice in the dark. The pellets were washed with 1X HBSS, incubated with 1% PFA and washed again with BB. The tubes were kept on ice until flow cytometry analyses. Blood flow cytometry was done on blood samples taken through intra-ventricular punction on the same animals before perfusion. Blood samples were placed in collection tubes coated with K2EDTA anticoagulant before performing the experiment. Samples were analyzed with BD LSRII cell analyzer, and data analyzed with FlowJo software (BD). Doublets were discriminated using forward scatter and side scatter (area and height).

### Quantification and statistical analysis

All obtained data were analyzed using Prism software (v9.0.1; GraphPad). Normality of the data distribution was first tested using a Shapiro-Wilk test and no outliers were removed from the study. Prism graphs were formatted using a boxplot to display the data. A non-parametric Kruskal-Wallis test was used to analyze the density of FLT3^+^ cells throughout life. The investigation of the morphology using fractal analysis was statistically analyzed using a paired student t-test. Statistical analysis of the TMEM119 and P2RY12 colocalization was done using a non-parametric student t-test with Welch’s correction. Across the statistical analysis, a threshold of *p* < 0.05 was considered as statistically significant (∗ = *p* < 0.05, ∗∗ = *p* < 0.01, ∗∗∗ = *p* < 0.001, ∗∗∗∗ = *p* < 0.0001).
