# Supplementary material for: Bone marrow-derived myeloid cells transiently colonize the brain during postnatal development and interact with glutamatergic synapses
Source: iScience. 2024 May 21;27(7):110037. doi: 10.1016/j.isci.2024.110037 (PMC11253522; doi:10.1016/j.isci.2024.110037)
Supplement: Document S1. Figures S1–S4 and Table S1 [file mmc1.pdf]

iScience, Volume *27*

Supplemental information

Bone marrow-derived myeloid cells transiently colonize the brain during postnatal development and interact with glutamatergic synapses

Micaël Carrier, Marie-Ève Robert, Marie-Kim St-Pierre, Fernando González Ibáñez, Elisa Gonçalves de Andrade, Audrée Laroche, Katherine Picard, Haley A. Vecchiarelli, Julie C. Savage, Éric Boilard, Michèle Desjardins, and Marie-Ève Tremblay

Supplemental Information

Graphical abstract: Summary of our findings on FLT3$^+$IBA1$^+$ cells infiltration and synaptic interaction in the healthy developing mouse brain.

FLT3$^+$IBA1$^+$ cells are illustrated in red and FLT3$^-$IBA1+ microglia are illustrated in green as seen on the manuscript fluorescence data. We observed FLT3$^+$IBA1$^+$ cells infiltrating the brain during the two first weeks of mouse life. These cells presented a unique morphology compared to microglia while similarly pruning glutamatergic synapses, hence contributing with microglia to mediating normal brain development.

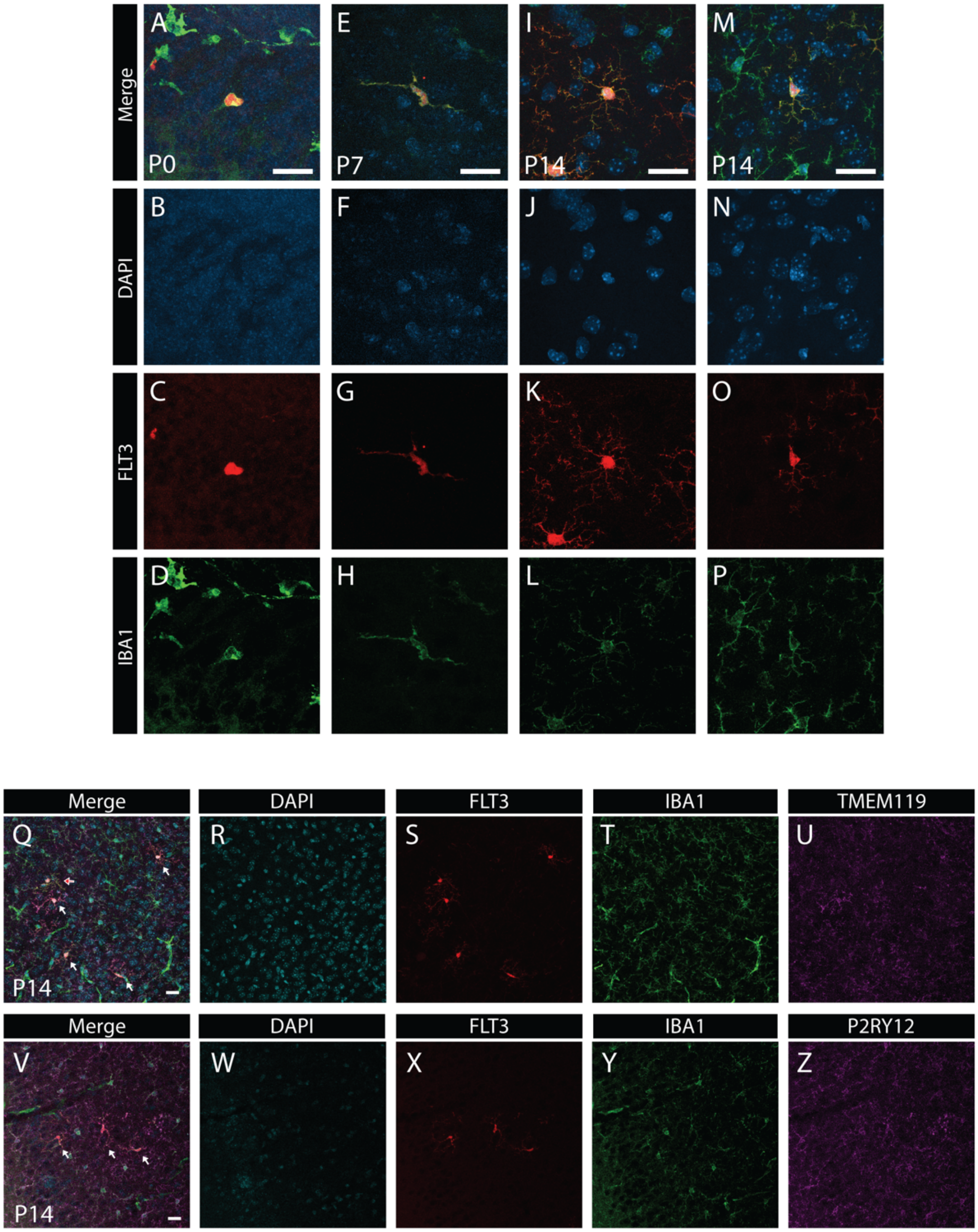

Supp. Figure 1 FLT3⁺IBA1⁺ cell morphology and markers expression in confocal microscopy. (A–P) Representative images of the FLT3⁺IBA1⁺ cells across ages. (Q-U) Representative image of the TMEM119 staining channels. (V-Z) Representative image of the P2RY12 staining channels, FLT3⁺IBA1⁺ cells are highlighted with white arrows.

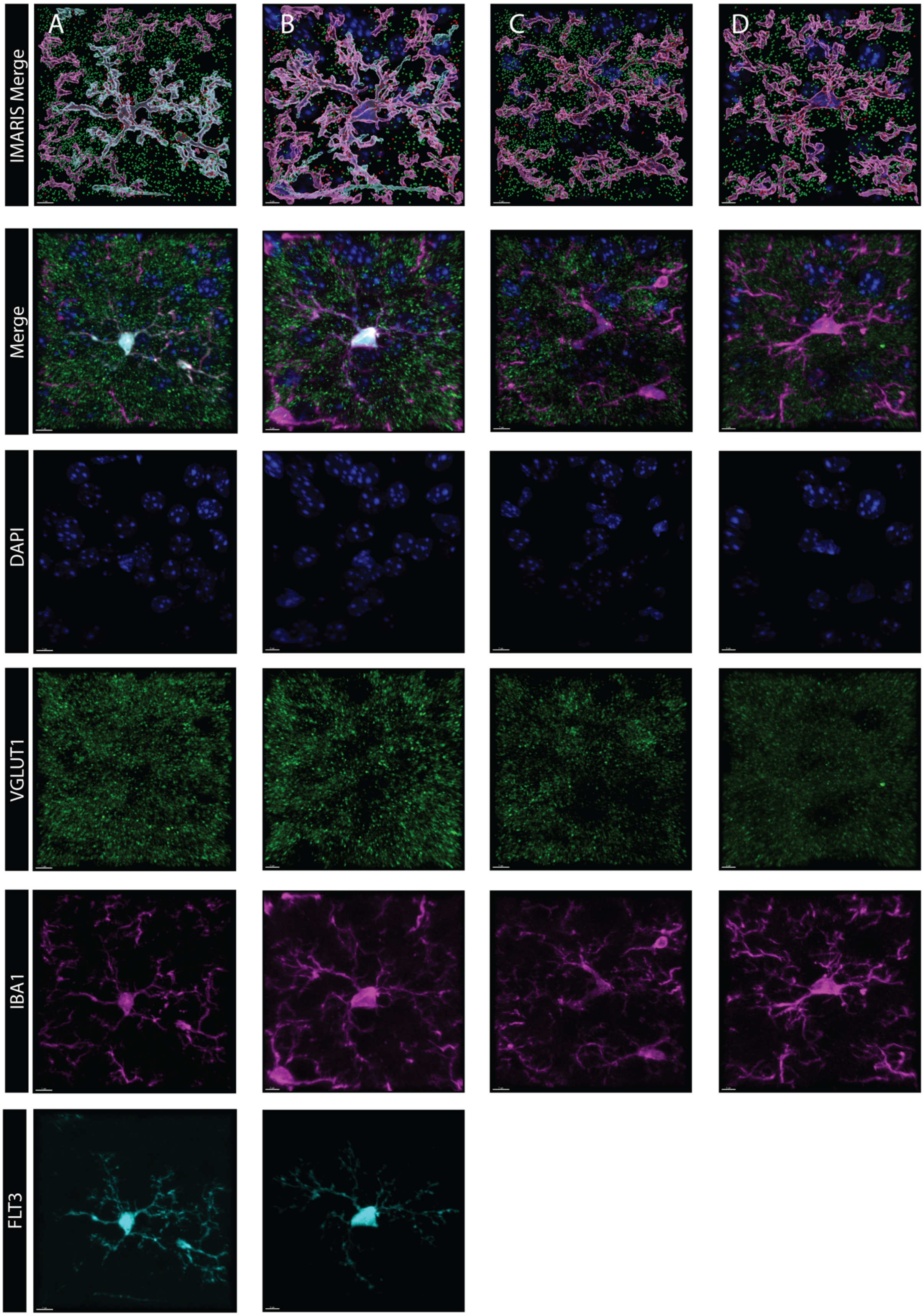
A
B
C
D
IMARIS Merge
Merge
DAPI
VGLUT1
IBA1
FLT3

Supp. Figure 2 FLT3⁺IBA1⁺ cell pruning of VGLUT1 synapses in confocal microscopy. (A–B) Split channel view of the VGLUT1 staining in FLT3⁺IBA1⁺ cells of the mouse motor cortex at P14. (C–D) Split channel view of the VGLUT1 staining of in FLT3⁻IBA1⁺ microglia in the mouse motor cortex at P14.

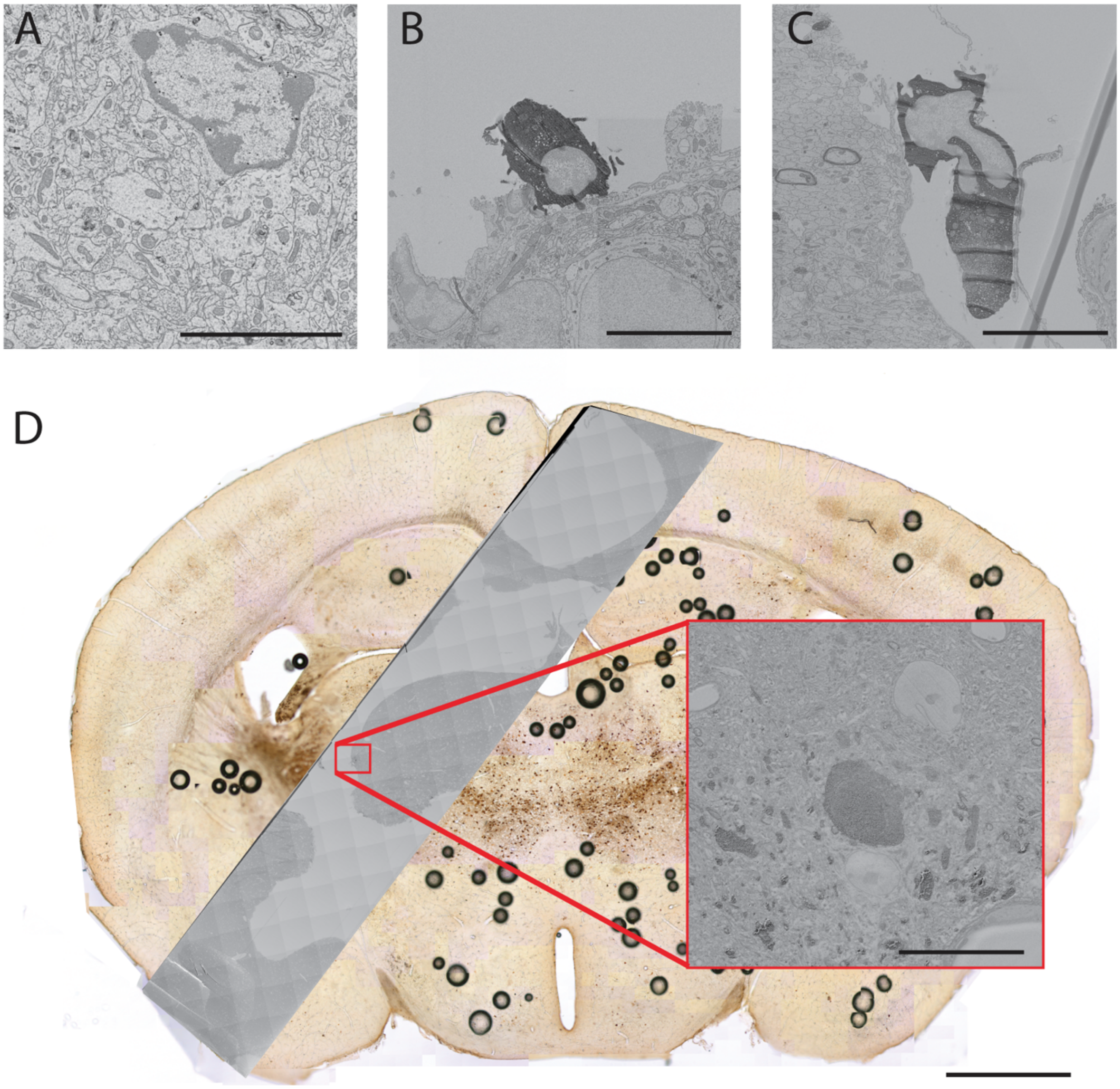

Supp. Figure 3 Mapping of the FLT3⁺ cells in electron microscopy. (A) Typical microglia found in the P14 piriform cortex. (B–C) Peripheral BMDC in the circulation stained with the anti-RFP antibody. (D) Correlative experiment where the mouse brain free-floating section was scanned using brightfield microscopy before electron

microscopy processing, inset of a FLT3^{+} cell found in the region scanned in electron microscopy.

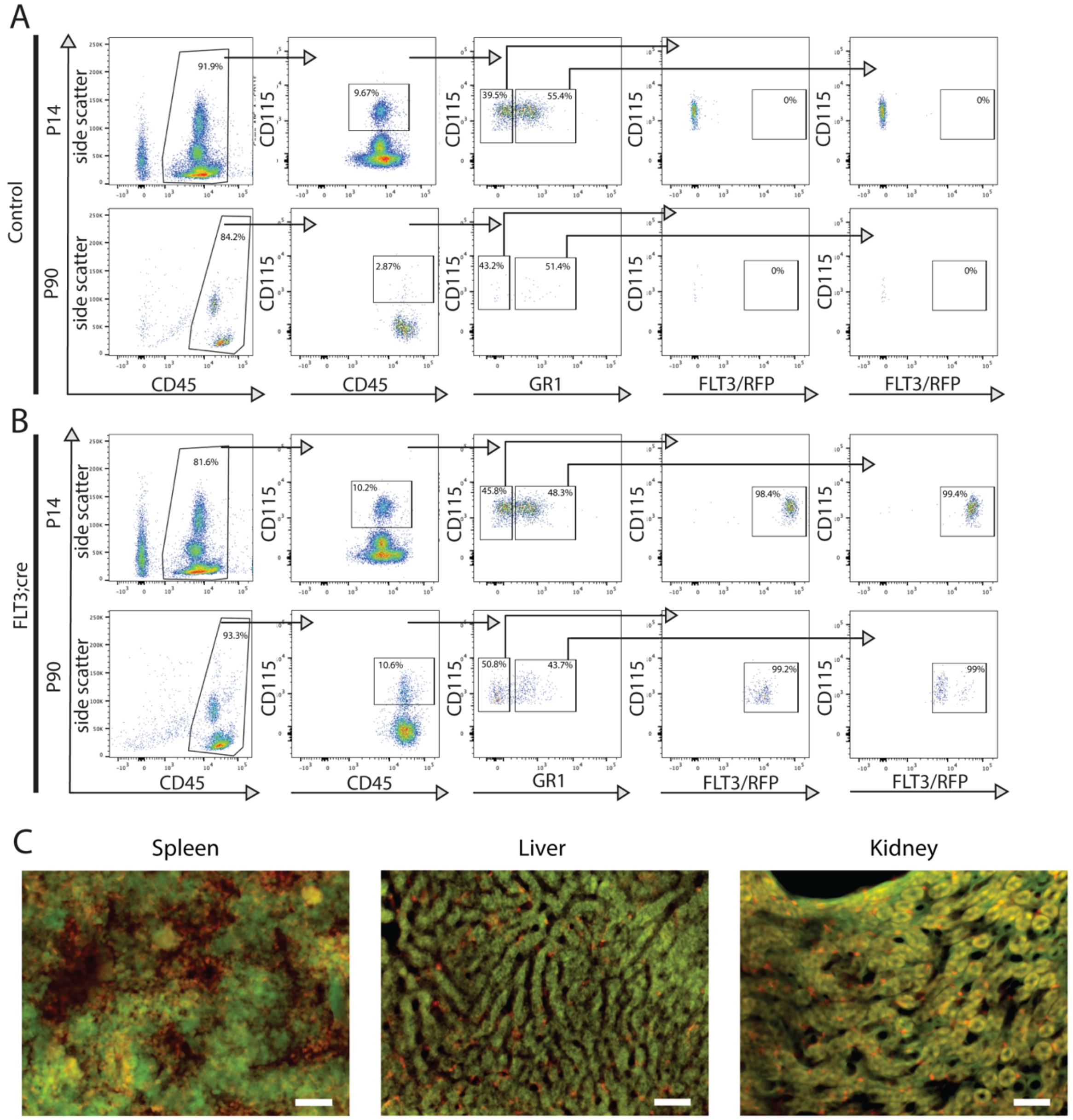

Supp. Figure 4 Effectiveness of the model: FLT3^{+} cells in the periphery strongly express red fluorescent protein in the Flt3;cre; RFP-Flox model. (A) Representative flow cytometry analysis of the FLT3^{+} cells in the control P14 and P90 mouse blood (N=1 animals). (B) Representative FACs analysis of the infiltrating FLT3^{+} cells in the FLT3;cre P14 and P90 mice blood (realized on two P14 and six P90 animals). Numbers represent percentage from parent gate (arrow). (C) Representative picture of FLT3^{+} cells in different FLT3;cre animal organs (spleen, liver, kidney), Scale bar = 50 mm.

Supp. Table 1 Condition description for the fluorescence staining for IBA1, TMEM119, P2RY12 and VGLUT1 realized in this study.

Antibody	Animal	Imaging	Antigen retrieval	Quenching	Blocking	Primary antibody	Secondary antibody	Nuclear counterstain	Primary antibody #2	Secondary antibody #2
IBA1	FLT3^{cre}-RFP^{lox} P14, P90	Slide scanner	Citrate buffer at 70ºC for 40 min	0,1% NaBH4 (in PBS) for 30 min	5% NGS, 5% NDS, 0,5% gelatin, 0,01% Triton (in	1:150 in blocking buffer	1:300 donkey anti-mouse in blocking buffer	1:10000 DAPI for 5 min		
IBA1	FLT3^{cre}-RFP^{lox} P14	Confocal	Citrate buffer at 70ºC for 40 min	0,1% NaBH4 (in PBS) for 30 min	5% NGS, 5% NDS, 0,5% gelatin, 0,01% Triton (in	1:150 in blocking buffer	1:300 donkey anti-mouse in blocking buffer	1:10000 DAPI for 5 min		
IBA1 and TMEM119 (cocktail)	FLT3^{cre}-RFP^{lox} P14	Confocal	Citrate buffer at 70ºC for 40 min	0,1% NaBH4 (in PBS) for 30 min	5% NGS, 5% NDS, 0,5% gelatin, 0,01% Triton (in	IBA1 1:150 and TMEM119 1:300 in	1:300 donkey anti-mouse Alexa 488 and 1:300 goat anti-	1:10000 DAPI for 5 min		
IBA1 and P2Y12 (cocktail)	FLT3^{cre}-RFP^{lox} P14	Confocal	Citrate buffer at 70ºC for 40 min	0,1% NaBH4 (in PBS) for 30 min	5% NGS, 5% NDS, 0,5% gelatin, 0,01% Triton (in	IBA1 1:150 and P2RY12 1:500 in blocking buffer	1:300 donkey anti-mouse Alexa 488 and 1:300 goat anti-	1:10000 DAPI for 5 min		
VGLUT1 (ab#1) and IBA1-635 (ab#2)	FLT3^{cre}-RFP^{lox} P14	Confocal	No antigen retrieval	0,1% NaBH4 (in PBS) for 30 min	5% NGS, 0,5% gelatin, 0,3% Triton (in PBS) for 1	1:500 in blocking buffer	1:300 goat anti-rabbit in blocking buffer	1:10000 DAPI for 5 min	1:100 in blocking buffer without triton	IBA1-635 is already conjugated
